# Hormonal-Receptors-Positive and HER2-Negative Patients with Metastatic Breast Cancer Treated with First-Line Palbociclib and Hormonal Therapy: Impact of First-Cycle Neutropenia and Dose Reduction on Therapeutic Outcome

**DOI:** 10.1155/2023/8994954

**Published:** 2023-08-25

**Authors:** Khaled Abd Elaziz Ahmed Elnaghi, Hosam Ali Alghanmi, Shereef Ahmed Elsamany, Fathia Almarzoki, Mohamed Elsafty, Mohammad Jaffal

**Affiliations:** ^1^Medical Oncology Department, Oncology Center, King Abdullah Medical City, Makkah, Saudi Arabia; ^2^Medical Oncology, Oncology Center, Mansoura University, Mansoura, Egypt; ^3^Department of Pharmacy, King Abdullah Medical City, Makkah, Saudi Arabia

## Abstract

**Background:**

CDK 4/6 inhibitors with hormonal therapy are the standard first-line therapy in metastatic hormonal receptors (HR)-positive and HER2-negative breast cancer. This study aims to assess the impact of neutropenia with 1st cycle, dose reduction, HER2-low status, and other clinicopathological factors on survival outcomes with the first-line palbociclib and hormonal therapy. *Patients and Methods*. In this retrospective study, we recruited patients with metastatic HR-positive and HER2-negative breast cancer. Neutropenia with 1st cycle, palbociclib dose reduction in addition to different clinicopathological and survival data were checked in patients' medical records. Survival outcomes were compared according to the abovementioned factors.

**Results:**

We recruited 150 patients who received first-line palbociclib with hormonal therapy. 86% of patients developed 1st cycle neutropenia which was more common in patients with high Ki67. Dose reduction was recorded in 46.7% of patients and it was more common in patients with higher Allred scores (scores 7–8). The median progression-free survival (PFS) of the study group was 22 months. No significant difference was observed in PFS according to the 1st cycle of neutropenia or grade of neutropenia. Similarly, no difference in PFS according to palbociclib dose reduction and HER2 low status was observed. Only the Allred score and having a single site of metastasis had an independent significant relation with PFS. The median overall survival (OS) of the study group was 39 months. No significant difference was observed in OS according to the 1st cycle neutropenia, grade of neutropenia, palbociclib dose reduction, and HER2-low status. Only the Allred score and having a single site of metastasis had an independent significant relation with OS. In addition, no difference was observed in PFS and OS according to ECOG PS (2 vs. 0-1) or menopausal status.

**Conclusion:**

No significant impact of the 1st cycle neutropenia, dose reduction, having ECOG PS2, menopausal status, or HER2 low status on survival outcome was observed. Survival outcome was significantly better in patients with single metastatic sites and higher ER-Allred scores.

## 1. Background

Endocrine therapy is a cornerstone in the management of hormonal receptors (HR)-positive and HER2-negative breast cancer [1]. However, de novo and acquired endocrine resistance remains a substantial problem as up to 30% of HR-positive breast tumors do not respond to endocrine therapy. Several mechanisms of resistance to endocrine treatment have been elucidated and recent therapeutic strategies to overcome resistance were approved for clinical use [2].

CDK 4/6 generates a complex with cyclin D, which in turn allows for the phosphorylation and inactivation of the retinoblastoma protein (Rb), a tumor suppressor protein, enabling gene transcription [3]. Ultimately, this results in the progression of a cell from the G1 to the S phase of the cell cycle and eventually leads to cell division [3]. CDK 4/6 inhibitors are a novel class of therapeutics that specifically target CDK 4/6 and block the phosphorylation of Rb [4, 5].

Several randomized clinical trials have confirmed that the addition of CDK4/6 inhibitors to hormonal therapy is a valuable clinical approach [6–9]. Three CDK4/6 inhibitors have now been approved for the treatment of ER-positive metastatic breast cancer: palbociclib, ribociclib, and abemaciclib [6–9].

Note that the outcome of patients who receive first-line CDK4-6 inhibitors with hormonal therapy is variable and there are no established biomarkers to predict the benefit of this therapy apart from ER-positivity [6]. HER2-low status recently emerged as a distinct entity which is the target of a new class of antibody-drug conjugate (ADC) such as trastuzumab deruxtecan. It is still unclear whether there is a differential outcome with CDK4-6 inhibitors in HER2-low vs. HER2-negative patients [10].

Neutropenia is the most common side effect of CDK4-6 inhibitors which is more common with palbociclib/ribociclib compared to abemaciclib. Moreover, dose reduction is encountered in about one-third of the patients [6–9]. In addition, the landmark studies recruited patients with ECOG performance status (PS) (0-1). Patients with PS2 were not represented in clinical trials [6–9].

The present study aims to assess the impact of the development of neutropenia (with 1st cycle) and dose reduction in addition to different clinicopathological factors such as ECOG PS and HER2 low status, on the outcome of the first-line therapy of palbociclib with hormonal therapy in HR-positive/HER2-negative metastatic breast cancer patients.

## 2. Materials and Methods

### 2.1. Study Design

In this retrospective study, patients with HR-positive and HER2-negative metastatic breast cancer, who received palbociclib with hormonal therapy in the first line, were recruited. Neutropenia with 1st cycle, palbociclib dose reduction in addition to different clinicopathological and survival data were checked in patients' medical records. Neutropenia is described as an abnormally low number of neutrophils in blood graded as grade 1 (1500–2000), grade 2 (>1500−1000), grade 3 (<1000−500), and grade 4 (<500). Survival outcomes were compared according to the abovementioned factors. Progression-free survival (PFS) is defined as the time from the date of starting palbociclib with hormonal therapy till the date of the first disease progression or death (any cause). Overall survival (OS) is defined as the time from the date of starting palbociclib with hormonal therapy to the date of patient death, due to any cause, or to the last date at which the patient was known to be alive.

### 2.2. Study Population

We recruited patients with histologically confirmed invasive breast carcinoma with HR-positive and HER2-negative phenotype and clinical or radiological evidence of metastasis. Patients should have ECOG PS 0–2 and received the first-line therapy with palbociclib in addition to hormonal therapy. This treatment should have started between 1/1/2018 and 31/12/2020. Only patients with complete medical records were included.

### 2.3. Assessment Methods and Procedures

Eligible patients were checked through hospital medical records. For each patient the following data were collected: age and menopausal status at diagnosis, date of relapse after an early breast cancer diagnosis, if applicable, date of diagnosis of metastatic disease, site(s) of metastasis, ER status, percentage and staining intensity, Allred's score, PR-status, percentage and staining intensity, Ki67% (by manual method), and HER2 status by immunohistochemistry (IHC) with FISH confirmation in cases with (++) by IHC. HER2 low status was defined as HER-2 +1 or HER-2 +2 with a negative FISH. In addition, treatment-related data were collected including the type of hormonal therapy used with palbociclib, neutropenia after the first treatment cycle of palbociclib with hormonal therapy, and grade of neutropenia in addition to palbociclib dose reduction (if any). The date of progression after the first-line systemic therapy with palbociclib and hormonal therapy in addition to the date of death (if applicable) was checked.

### 2.4. Statistical Analysis

SPSS version 17 was used for statistical analysis. Categorical data were presented as percentages and differences in the distribution of categorical variables according to factors of interest which were assessed using the chi-square test. Survival data were presented by the Kaplan–Meier method and differences in survival were evaluated using the log-rank test. Factors with significant survival benefits in univariate analysis were assessed in multivariate analysis using the Cox regression method. As a general rule, an alpha value of 0.05 will be set as a priori for all two-tailed comparisons.

## 3. Results

### 3.1. Baseline Characteristics

We recruited 150 patients who were eligible for the study within the specified timelines. All patients were ER-positive, 84.7% were PR-positive, 44.6% were HER2-low, and 40% had Ki67 >20%. In addition, 52% were premenopausal, 26% had ECOG PS2, and 64% had stage IV disease at the time of diagnosis.

Note that 86% of patients developed neutropenia after the first cycle. We assessed different clinicopathological factors in those who developed neutropenia after the 1st cycle compared to those without neutropenia. First-cycle neutropenia was more common in patients with high Ki67 than in patients with low Ki67 (93.3% vs. 82.6%, *p*=0.*β*02). However, other factors were balanced between the two groups (Table 1).

Dose reduction was recorded in 46.7% of patients. Dose reduction was more common in patients with higher Allred scores (scores 7–8) than in patients with lower Allred scores (score ≤ 6) (50.0% vs. 29.2%, respectively, *p*=0.048). No difference in dose reduction level according to ECOG PS (0-1 vs. 2) or menopausal status was found. Similarly, no significant difference was observed in dose reduction according to other factors (Table 2).

Meanwhile, regarding HER2-low status, no significant difference was found according to Ki67 level, Allred score, PR-status, or other clinicopathological factors (Table 3).

### 3.2. Survival Outcome

**Figure 1:**
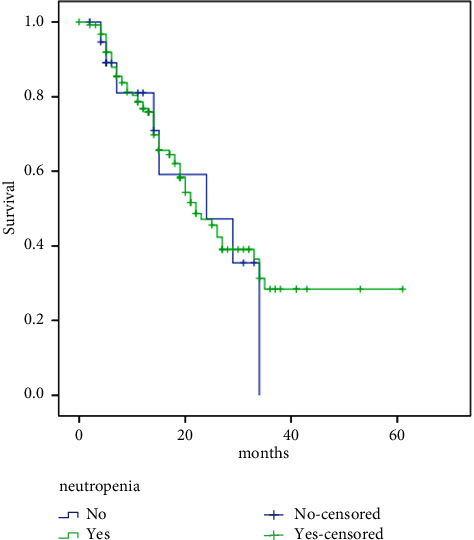
Neutropenia and survival relation.

With a median follow-up of 23 months, the median PFS of the study group was 22 months. No significant difference in PFS in those with neutropenia compared to patients who did not develop neutropenia with the first cycle (yes: 22.0 vs. no: 24 months, HR 1.13, 95% CI: 0.54–2.36, *p*=0.75), (Figure 1) was observed.

**Figure 2:**
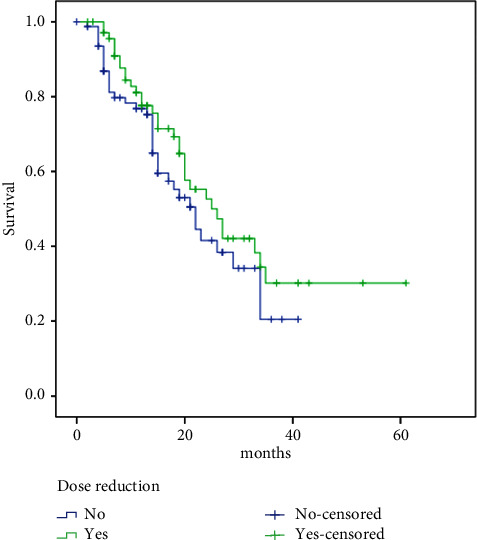
Dose reduction and survival relation.

No difference in PFS as well as according to the grade of neutropenia (GIII/IV: 23 months vs. GI/II: 22 months, HR 0.76, 95% CI: 0.41–1.41, *p*=0.39) was observed. Similarly, no difference was observed in PFS according to palbociclib dose reduction (no: 22 months vs. yes: 25 months, HR 1.33, 95% CI: 0.83–2.14, *p*=0.23), (Figure 2) and HER2 low status (no: 22 months vs. yes: 24 months, HR 1.03, 95% CI: 0.64–1.64, *p*=0.92).

Interestingly, no significant difference was observed in PFS according to menopausal status (premenopausal: 22 months vs. postmenopausal: 25 months, HR 1.11, 95% CI: 0.69–1.77, *p*=0.67) and in those with PS2 compared to PS 0–1 (PS2: 21 months vs. PS 0–1: 23 months, HR 1.097, 95% CI: 0.65–1.85, *p*=0.73).

**Figure 3:**
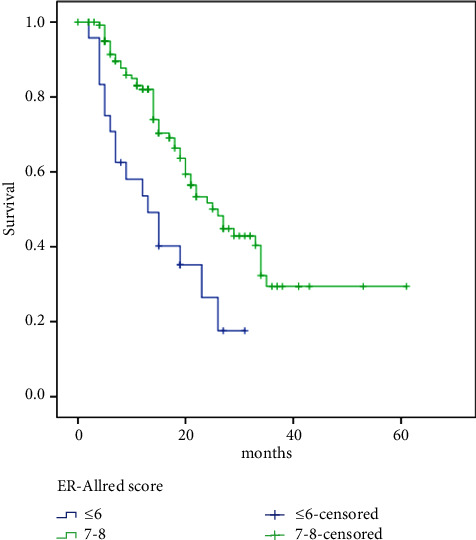
ER Allred score and progression-free survival relation.

Meanwhile, PFS was significantly better in patients with higher Allred scores (low: 13 vs. high: 25 months, HR 2.42, 95% CI: 1.39–4.23, *p*=0.002) (Figure 3), low Ki67 (low: 26 vs. high: 19 months, HR 0.63, 95% CI: 0.39–1.02, *p*=0.058), and patients with PR-positive tumors (negative: 15 vs. positive: 25 months, HR 1.96, 95% CI: 1.07–3.62, *p*=0.03), bone-only metastasis compared with other sites (bone-only: 35 vs. others: 20 months, HR 0.45, 95% CI: 0.23–0.88, *p*=0.02), and single vs. multiple sites of metastases (single: 38 vs. multiple: 18 months, HR 0.34, 95% CI: 0.19–0.59, *p* < 0.00001).

The multivariate analysis shows that among variables such as Allred score, PR-status, Ki67 level, bone-only metastasis, and number of metastatic sites (single vs. multiple), only Allred score (*p*=0.015) and having a single site of metastasis (*p*=0.006) had an independent significant relation with PFS (Table 4).

**Figure 4:**
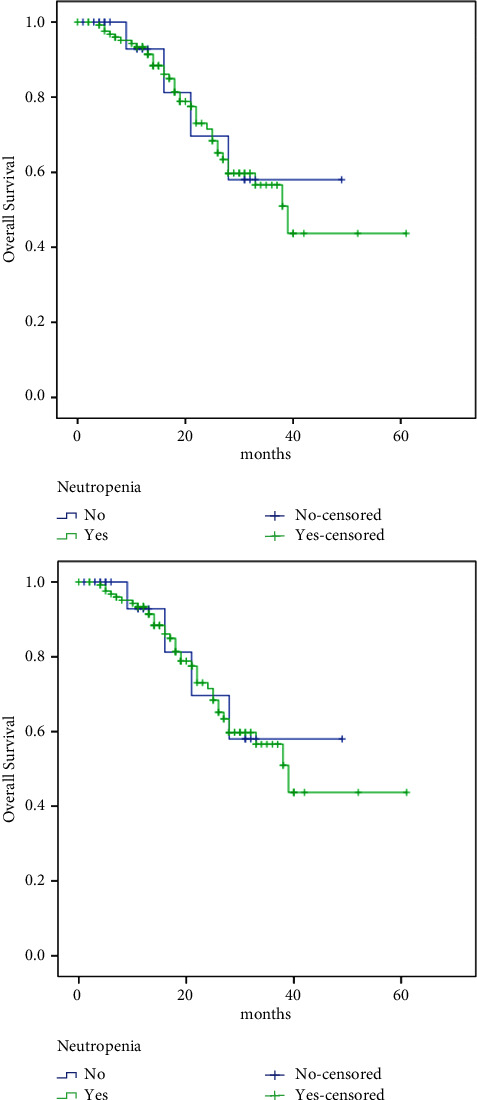
Neutropenia and survival relation.

The median OS of the study group was 39 months. No significant difference in OS in those without neutropenia compared to patients who developed neutropenia with the first cycle (no: NR vs. yes: 39.0 months, HR 0.93, 95% CI: 0.33–2.61, *p*=0.89) (Figure 4) was observed.

No difference in OS as well as according to the grade of neutropenia (GIII/IV: 23 months vs. GI/II: 22 months, HR 0.76, 95% CI: 0.41–1.41, *p*=0.39) was observed. Similarly, no difference in OS according to HER2 low status (yes: 38 months vs. no: 39 months, HR 1.01, 95% CI: 0.54–1.89, *p*=0.97) was observed.

Interestingly, no significant difference in OS according to menopausal status (premenopausal: 39 vs. postmenopausal: 40 months, HR 1.17, 95% CI: 0.62–2.17, *p*=0.63), Ki67 level (low: 39 vs. high: 28 months, HR 0.55, 95% CI: 0.29–1.036, *p*=0.064), and in those with PS2 compared with PS 0–1 (PS 0–1: 39 months vs. PS2: 33 months, HR 0.83, 95% CI: 0.53–1.62, *p*=0.59) was observed.

**Figure 5:**
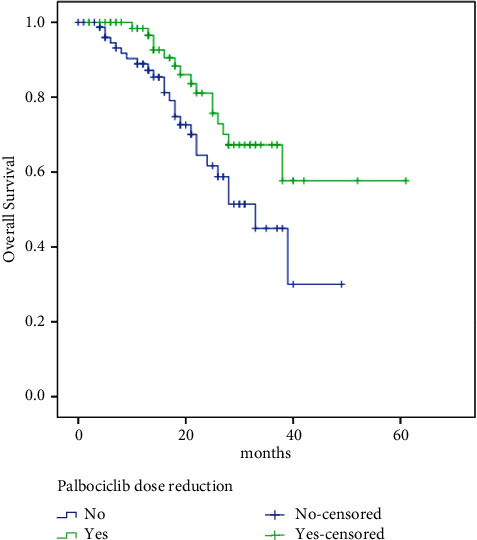
Palbociclib dose reduction and survival relation.

**Figure 6:**
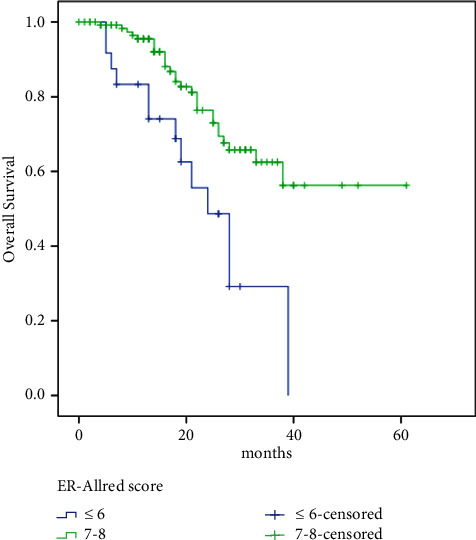
ER Allred score and survival.

**Figure 7:**
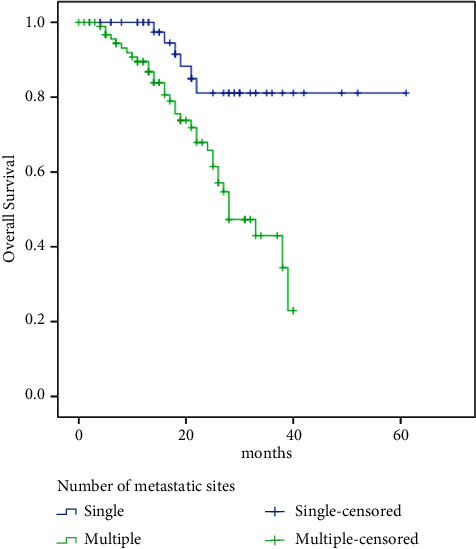
Survival in a single metastatic site versus multiple sites.

Meanwhile, OS was significantly better in patients with palbociclib dose reduction (no: 33 months vs. yes: NR, HR 1.96, 95% CI: 1.032–3.73, *p*=0.04) (Figure 5), higher Allred score (low: 24 months vs. high: NR, HR 2.81, 95% CI: 1.44–5.46, *p*=0.002) (Figure 6), and in patients with PR-positive tumors (negative: 22 vs. positive: 39 months, HR 2.31, 95% CI: 1.09–4.91, *p*=0.029), bone-only metastasis compared with other sites (bone-only: NR vs. others: 28 months, HR 0.29, 95% CI: 0.11–0.85, *p*=0.024), and single vs. multiple sites of metastases (single: NR vs. multiple: 18 months, HR 0.25, 95% CI: 0.11–0.60, *p* < 0.00001) (Figure 7).

The multivariate analysis shows that among variables such as Allred score, PR-status, bone-only metastasis, number of metastatic sites (single vs. multiple), and palbociclib-dose reduction, only having a single site of metastasis (0.04) and Allred score (*p*=0.026) had an independent significant relation with OS (Table 5).

## 4. Discussion

CDK4-6 inhibitors with hormonal therapy are currently the standard of care in the first-line therapy of advanced HR-positive and HER2-negative breast cancer. A coupling of toxicity with treatment efficacy has been reported with several medications. This provides the rationale to explore the outcome of first-line palbociclib with hormonal therapy according to the development of neutropenia, grade of neutropenia, and palbociclib-dose reduction.

Neutropenia is the most common side effect of this class. Neutropenia was reported in 86% of patients in our study which is comparable to palbociclib data reported in PALOMA 2 trial. In PALOMA 2 trial, the most common hematological toxicity is neutropenia followed by leukopenia accounting for around 79% and 39%, respectively [9]. Note that, in our analysis, neutropenia was more frequent in patients with higher Ki67 levels which may be explained by the increased palbociclib activity in patients with rapidly proliferating tumors, which may be translated to more drug-related toxic effects.

Dose reduction was reported in about one-third of the cases in clinical trials which is commonly associated with grade 3–4 hematological toxicity and diarrhea [11]. Dose reduction in our study occurred in around 46% of patients, which is higher than that reported in other real-world data. In real-world data from the USA, dose reduction was reported in 20.1% of patients after a median of six cycles [12], while in another report from the Netherlands, 33% had a dose reduction [13]. However, these studies included a higher number of patients (763 and 598 patients, respectively) compared with 150 patients only in our study. Meanwhile, the higher frequency of palbociclib-dose reduction in our study may be explained by several factors such as including patients with ECOG PS2 and patients receiving previous chemotherapy (47%). However, it is not clear if racial factor plays a role in this context.

Note that, survival outcomes reported in our study are comparable with that reported in clinical trials [9] pointing out that a higher rate of dose reduction in this study did not impact therapeutic outcomes. This may be reassuring that dose reduction when indicated does not compromise treatment efficacy. A similar finding was reported in the abovementioned Dutch study with no impact of dose reduction on clinical outcomes [13].

In our retrospective study, around 26% of patients had ECOG PS2. Those patients were almost excluded from clinical trials [9]. We reported no difference in survival outcome compared to patients with ECOG PS 0–1. Similarly, no difference in the frequency of neutropenia or dose reduction according to PS status was observed. Our findings are consistent with the results of the systemic review of CDK4/6 inhibitors which showed similar PFS and OS in patients with ECOG PS2 compared with PS 0–1 [14]. This may point to the feasibility of palbociclib with hormonal therapy in patients with ECOG PS2.

Recently, there is growing interest on the HER2-low category of breast cancer. This category derives a clinically meaningful benefit from new ADCs such as trastuzumab deruxtecan [15, 16]. In our study, no difference in palbociclib/hormonal therapy outcome in HER-2- low compared with HER2-negative patients was observed. However, the outcome of HER2-low breast cancer with CDK4/6 inhibitors is conflicting and not consistent in the available reports. In a retrospective analysis of 186 patients with Her2-low breast cancer who received CDK4/6 inhibitors in a single institution, there was no difference in survival outcome compared with HER2-negative patients [17]. However, in another retrospective study including 106 women, PFS was inferior in HER2-low compared with HER2-negative patients (8.9 vs. 18.8 months, respectively, *p*=0.014) [15]. This may raise the importance to have data reported from a higher number of patients to elucidate the exact outcome of HER2-low breast cancer with CDK4-6 inhibitors.

In concordance with the results of PALOMA 3 and MONARCH 2 [18, 19], we reported similar survival outcomes in premenopausal and postmenopausal patients. These studies included a subgroup of premenopausal patients who had survival benefits from CDK 4–6 inhibitors with hormonal therapy compared with hormonal therapy alone, which is comparable to the benefit in postmenopausal patients. This finding was confirmed in a meta-analysis including 9 phase 2 and 3 studies which reported similar benefits from CDK4-6 inhibitors in both premenopausal and postmenopausal patients [11].

In the current study, patients with solitary sites of metastasis had significantly better PFS and OS compared to those with multiple sites of metastases. Note that, in PALOMA 2 study, the benefit of palbociclib was consistent in patients with single and multiple sites of metastasis [9]. Similarly, in MONALEESA 3 study, the survival benefit of ribociclib was consistent irrespective of the number of metastatic sites (<3 vs. ≥3 sites) [20]. More prominent survival benefits in patients with a single metastatic site in our report (with no comparator arm) may be explained by the better prognostic outcome in those patients.

In the current report patients with higher ER-Allred scores (who are more endocrine sensitive), had better survival outcomes than those with lower scores (Allred score 7–8 vs. ≤6). In the subgroup analysis of the PALOMA 3 study, the OS benefit of adding palbociclib to hormonal therapy was more pronounced in patients with sensitivity to previous endocrine therapy [18]. Nevertheless, accumulating data suggested that patients with a low expression of ER receptors (1%–10%) have a low response to hormonal therapy and behave biologically like triple-negative breast cancer [21].

This study has some limitations including a relatively small number of patients, lack of a control arm, and being a single institute study. However, the study provides real-world evidence of the activity and safety of first-line palbociclib which included some populations of patients not included in clinical practice such as patients with ECOG PS2.

In conclusion, the survival outcome with first-line therapy of palbociclib with hormonal therapy was comparable with previous reports of palbociclib in clinical trials. No significant impact of 1st cycle neutropenia, dose reduction, having ECOG PS2, menopausal status, or HER2 low status on survival outcome was observed. Meanwhile, survival outcome was significantly better in patients with single metastatic sites and higher ER-Allred scores.

## Figures and Tables

**Table 1:** Baseline clinicopathological factors and relation to neutropenia after 1^st^ cycle.

Parameters		Neutropenia		Total	*P* value
		No	Yes		
ECOG PS	0	0	2	2	0.82
		0.0%	100.0%	100.0%	
	1	15	94	109	
		13.8%	86.2%	100.0%	
	2	6	33	39	
		15.4%	84.6%	100.0%	

BMI	Normal	2	21	23	0.71
		8.7%	91.3%	100.0%	
	Overweight	7	37	44	
		15.9%	84.1%	100.0%	
	Obese	12	71	83	
		14.5%	85.5%	100.0%	

Ki67 level	Low	15	71	86	0.02
		17.4%	82.6%	100.0%	
	High	4	56	60	
		6.7%	93.3%	100.0%	
	NA	2	2	4	
		50.0%	50.0%	100.0%	

Menopausal status	Premenopause	8	70	78	0.17
		10.3%	89.7%	100.0%	
	Postmenopause	13	59	72	
		18.1%	81.9%	100.0%	

Low HER2 expression	No	10	73	83	0.44
		12.0%	88.0%	100.0%	
	Yes	11	56	67	
		16.4%	83.6%	100.0%	

Stage at diagnosis	Stage II	1	3	4	0.68
		25.0%	75.0%	100.0%	
	Stage III	8	42	50	
		16.0%	84.0%	100.0%	
	Stage IV	12	84	96	
		12.5%	87.5%	100.0%	

*N*-stage	*N*x	3	1	4	0.008
		75.0%	25.0%	100.0%	
	*N*0	20	70	90	
		22.2%	77.8%	100.0%	
	*N*1	5	59	64	
		7.8%	92.2%	100.0%	
	*N*2	4	30	34	
		11.8%	88.2%	100.0%	
	*N*3	2	9	11	
		18.2%	81.8%	100.0%	

(Neo) adjuvant chemotherapy	No	17	111	128	0.48
		13.3%	86.7%	100.0%	
	Yes	4	17	21	
		19.0%	81.0%	100.0%	

PR-status	Negative	4	19	23	0.61
		17.4%	82.6%	100.0%	
	Positive	17	110	127	
		13.4%	86.6%	100.0%	

Site of metastasis	Bone-only	5	27	32	0.76
		15.6%	84.4%	100.0%	
	Others	16	102	118	
		13.6%	86.4%	100.0%	

Site of metastasis	Single	8	46	54	0.83
		14.8%	85.2%	100.0%	
	Multiple	13	83	96	
		13.5%	86.5%	100.0%	

**Table 2:** Discussing baseline tumor factors and relation to hormonal therapy interruption in regard to palbociclib dose reduction.

Parameters		Palbociclib dose reduction		Total	*P* value
		No = 80	Yes = 70		
ECOG PS	0	1	1	2	0.49
		50.0%	50.0%	100.0%	
	1	55	54	109	
		50.5%	49.5%	100.0%	
	2	24	15	39	
		61.5%	38.5%	100.0%	

BMI	Normal	15	8	23	0.40
		65.2%	34.8%	100.0%	
	Overweight	24	20	44	
		54.5%	45.5%	100.0%	
	Obese	41	42	83	
		49.4%	50.6%	100.0%	

Ki67 level	Low	48	38	86	0.78
		55.8%	44.2%	100.0%	
	High	30	30	60	
		50.0%	50.0%	100.0%	
	NA	2	2	4	
		50.0%	50.0%	100.0%	

Menopausal status	Premenopause	39	39	78	0.39
		50.0%	50.0%	100.0%	
	Postmenopause	41	31	72	
		56.9%	43.1%	100.0%	

Allred score	≤6	17	7	24	0.048
		70.8%	29.2%	100.0%	
	7-8	63	63	126	
		50.0%	50.0%	100.0%	

Stage at diagnosis	Stage II	3	1	4	0.44
		75.0%	25.0%	100.0%	
	Stage III	29	21	50	
		58.0%	42.0%	100.0%	
	Stage IV	48	48	96	
		50.0%	50.0%	100.0%	

(Neo) adjuvant chemotherapy	No	70	58	128	0.54
		54.7%	45.3%	100.0%	
	Yes	10	11	21	
		47.6%	52.4%	100.0%	

PR-status	Negative	12	11	23	0.90
		52.2%	47.8%	100.0%	
	Positive	68	59	127	
		53.5%	46.5%	100.0%	

Site of metastasis	Bone-only	15	17	32	0.41
		46.9%	53.1%	100.0%	
	Others	65	53	118	
		55.1%	44.9%	100.0%	

Site of metastasis	Single	26	28	54	0.34
		48.1%	51.9%	100.0%	
	Multiple	54	42	96	
		56.3%	43.8%	100.0%	

HER2 low status	No	47	36	83	0.37
		56.6%	43.4%	100.0%	
	Yes	33	34	67	
		49.3%	50.7%	100.0%	

**Table 3:** Discussing baseline tumor factors and relation to hormonal therapy interruption in regard to HER2-low.

Parameters		HER2-low		Total	*P* value
		No	Yes		
ECOG PS	0	0	2	2	0.16
		0.0%	100.0%	100.0%	
	1	64	45	109	
		58.7%	41.3%	100.0%	
	2	19	20	39	
		48.7%	51.3%	100.0%	

BMI	Normal	11	12	23	0.56
		47.8%	52.2%	100.0%	
	Overweight	23	21	44	
		52.3%	47.7%	100.0%	
	Obese	49	34	83	
		59.0%	41.0%	100.0%	

Ki67 level	Low	45	41	86	0.56
		52.3%	47.7%	100.0%	
	High	35	25	60	
		58.3%	41.7%	100.0%	
	NA	3	1	4	
		75.0%	25.0%	100.0%	

Menopausal status	Premenopause	40	38	78	0.30
		51.3%	48.7%	100.0%	
	Postmenopause	43	29	72	
		59.7%	40.3%	100.0%	

Allred score	≤6	16	8	24	0.22
		66.7%	33.3%	100.0%	
	7-8	67	59	126	
		53.2%	46.8%	100.0%	

Stage at diagnosis	Stage II	2	2	4	0.32
		50.0%	50.0%	100.0%	
	Stage III	32	18	50	
		64.0%	36.0%	100.0%	
	Stage IV	49	47	96	
		51.0%	49.0%	100.0%	

(Neo) adjuvant chemotherapy	No	67	61	128	0.10
		52.3%	47.7%	100.0%	
	Yes	15	6	21	
		71.4%	28.6%	100.0%	

PR-status	Negative	15	8	23	0.30
		65.2%	34.8%	100.0%	
	Positive	68	59	127	
		53.5%	46.5%	100.0%	

Site of metastasis	Bone-only	18	14	32	0.91
		56.3%	43.8%	100.0%	
	Others	65	53	118	
		55.1%	44.9%	100.0%	

Site of metastasis	Single	33	21	54	0.29
		61.1%	38.9%	100.0%	
	Multiple	50	46	96	
		52.1%	47.9%	100.0%	

**Table 4:** Multivariate analysis PFS.

Parameters	HR	95% confidence interval		*P* value
		Lower boundary	Upper boundary	
Ki67 level	0.731	0.441	1.212	0.224
Allred score	2.073	1.152	3.732	0.015
PR-status	1.230	0.641	2.362	0.53
Site of metastasis	1.392	0.472	4.105	0.548
Site of metastasis (single)	0.275	0.109	0.692	0.006

**Table 5:** OS multivariate.

Parameters	HR	95% confidence interval		*P* value
		Lower boundary	Upper boundary	
Allred score	2.22	1.10	4.50	0.026
PR-status	1.69	0.78	3.69	0.81
Site of metastasis	1.64	0.26	8.17	0.67
Site of metastasis (single)	0.22	0.053	0.935	0.04
Palbociclib dose reduction	1.62	0.83	3.17	0.16

## Data Availability

The datasets generated during and/or analysed during the current study are available from the corresponding author upon request.
